# Discovery of Non-Peptidic Compounds against Chagas Disease Applying Pharmacophore Guided Molecular Modelling Approaches

**DOI:** 10.3390/molecules23123054

**Published:** 2018-11-22

**Authors:** Shailima Rampogu, Gihwan Lee, Ayoung Baek, Minky Son, Chanin Park, Amir Zeb, Sang Hwa Yoon, Suhyeon Park, Keun Woo Lee

**Affiliations:** Division of Life Science, Division of Applied Life Science (BK21 Plus), Plant Molecular Biology and Biotechnology Research Center (PMBBRC), Research Institute of Natural Science (RINS), Gyeongsang National University (GNU), 501 Jinju-daero, Jinju 52828, Korea; shailima.rampogu@gmail.com (S.R.); pika0131@naver.com (G.L.); ayoung@gnu.ac.kr (A.B.); minky@gnu.ac.kr (M.S.); chaninpark0806@gmail.com (C.P.); zebamir85@gmail.com (A.Z.); jsyoon0517@gmail.com (S.H.Y.); parksh1994@naver.com (S.P.)

**Keywords:** Chagas disease, *Trypanosome cruzi*, cruzipian, cysteine protease, molecular docking simulations, molecular dynamics simulations

## Abstract

Chagas disease is one of the primary causes of heart diseases accounting to 50,000 lives annually and is listed as the neglected tropical disease. Because the currently available therapies have greater toxic effects with higher resistance, there is a dire need to develop new drugs to combat the disease. In this pursuit, the 3D QSAR ligand-pharmacophore (pharm 1) and receptor-based pharmacophore (pharm 2) search was initiated to retrieve the candidate compounds from universal natural compounds database. The validated models were allowed to map the universal natural compounds database. The obtained lead candidates were subjected to molecular docking against cysteine protease (PDB code: 1ME3) employing -Cdocker available on the discovery studio. Subsequently, two Hits have satisfied the selection criteria and were escalated to molecular dynamics simulation and binding free energy calculations. These Hits have demonstrated higher dock scores, displayed interactions with the key residues portraying an ideal binding mode complemented by mapping to all the features of pharm 1 and pharm 2. Additionally, they have rendered stable root mean square deviation (RMSD) and potential energy profiles illuminating their potentiality as the prospective antichagastic agents. The study further demonstrates the mechanism of inhibition by tetrad residues compromising of Gly23 and Asn70 holding the ligand at each ends and the residues Gly65 and Gly160 clamping the Hits at the center. The notable feature is that the Hits lie in close proximity with the residues Glu66 and Leu67, accommodating within the S1, S2 and S3 subsites. Considering these findings, the study suggests that the Hits may be regarded as effective therapeutics against Chagas disease.

## 1. Introduction

American Trypanosomiasis or Chagas disease is one of the leading causes of death in Latin America accounting to about 20,000~50,000 lives annually [1,2,3] World Health Organization (WHO) has labelled this disease as the neglected tropical disease, claiming approximately about 25 million lives at risk [4]. The disease is caused by the parasite *Trypanosoma cruzi* (*T. cruzi*) as a result of the blood meal from its human host by the insect and offering the parasite into the wound caused [3,5]. After which, the parasite penetrates into the cells of the host where it replicates, maturates and liberates the new progeny into the blood stream [6]. The primary cause of the disease transmission is through the insect and is additionally transmitted through transfusion of the infected blood and congenitally [7]. Broadly, the life cycle of *T. cruzi* can be classified into two replicative forms and two non- infective replicative forms. The former includes the epimastigote and amastigote while the latter is the metacyclic trypomastigote present in the insect and trypomastigote manifested in the blood of the infected humans [8,9] causing Chagas disease characterized by acute phase and the chronic phase [3,10]. The infection triggers when the infective forms of trypomastigotes outstretch to the bold stream thereby invading a host of cell types [7]. Later after the invasion the, trypomastigotes differentiates into amastigotes and rapidly replicates prior to the formation of trypomastigotes. Upon the destruction of the host cells, bloodstream trypomastigotes apparently spread by invading the other cells and can also be taken as a blood meal by the insect vector. Upon reaching the gastrointestinal tract of the insect, the trypomastigotes transform into the replicative epimastigotes, subsequently resulting into the metacyclic trypomastigotes. This process is also referred to as metacyclogenesis that occur towards the posterior edge of the insect gut eventually completing the life cycle [7].

The acute phase is prevalent for a short term, approximately two months and is generally symptom free or with more general non Chagas symptoms such as fever, body aches, vomiting, poor appetite, rashes, head ache and diarrhea [11]. However, some studies demonstrated the presence of hepatosplenomegaly, lymphadenopathy, presence of myocardidtis, platelet aggregation, spasm, vasculitis of coronary microcirculation and fibrin microthrombi formation [10,12,13,14]. Unlike the acute phase, the chronic phase displays its symptoms apparently much latter after the infections about 10~20 years and affect the heart, nervous system and the digestive system [11]. Additionally, the reduction in parasitemia is noticed due to the activation of acquired immunity [10] and is characterized by dilated cardiomyopathy [3].

Taken together, there is a dire need for addressing this problem that can combat the disease successfully. Of the several treatments given, some of the drugs were reported to be toxic and others developed drug resistance imparting no therapeutic activity [3,15,16,17,18,19]. Furthermore, reports exists that these drugs are effective in the acute phase and are ineffective in the patients with chronic infection [20]. Therefore, the discovery of the new drugs with reduced toxicity and high efficacy is of extreme importance.

Cysteine proteases often called as Cruzain [21,22] and sometimes referred to as cruzipian [23,24,25] illuminates to be a promising target for identifying new drugs. This belongs to the C1 protein family and is expressed at all the stages of *T. cruzi* playing an important role in cell invasion, immune invasion, intracellular multiplication and differentiation [4,26,27,28,29]. Over expression of Cruzain favors the formation of the infective form and the reduction of its activity averts the infection [17,26]. Furthermore, protease inhibitors have shown to hinder the replication and differentiation [30,31] besides acting efficiently by rendering positive results in mouse and dog models [32]. Additionally, it is well established that the cysteine protease inhibitors exert trypanocidal activity associated with negligible mammalian toxicity [4,33].

The first molecular structure of parasitic Cruzain was elucidated by McGrath et al. [21] that consisted of a single polypeptide chain with 215 amino acid residues represented by two domains, the α-helical (L domain) and antiparallel β-sheet (R domain). The catalytic triad comprising of Cys25, His159 and Asn175 residues and the substrate-binding site are located at the cleft between two domains. The L domain encompasses the residues from 12 to 112 and 208 to 212 with helical regions 25 to 40, 50 to 56 and 68 to 78. The R domain incorporates the residues from 1 to 11 and 113 to 207 with residues 117 to 127 and 139 to 142 as helices. Notably in Cruzain, the residue Gln19 is positioned to aid in catalysis [21].

The active site of Cruzain cysteine protease exhibits seven subsites, four toward the acyl side and three towards the amino acid side. The two domains of mature cysteine protease resembles a closed book with a front spine [34] projecting a V-shaped cavity at the top. Two residues Cys25 and His159 representing from each domain demonstrate the active catalytic site of the enzyme. Furthermore, the right (R-) domain resembles a β barrel imbibed with a short α helical motif, while the left (L-) domain comprising of about 30 residues forms the central core α helix [34]. Additionally, the ligands that occupy the subsites S1, S2 and S3 were considered to be more promising [11].

The objective of the present study is to discover new scaffolds as Cruzain inhibitors using various computational approaches that specifically interact with the catalytic residues together being locked at the S1, S2 and S3 subsites.

## 2. Results

### 2.1. Generation of the Pharmacophore Model

#### 2.1.1. Ligand-Based Pharmacophore Model Generation (Pharm 1)

**Figure 1:**
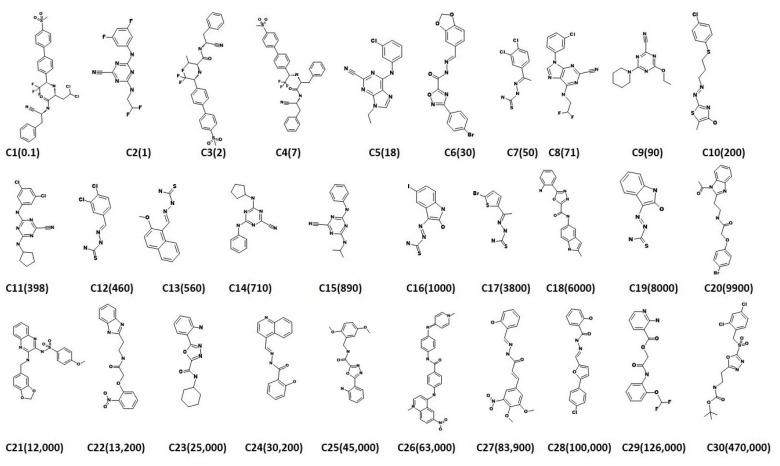
2D structural representation of 30 training set compounds employed for pharmacophore generation. Their experimental IC_50_ (nmol/L) values are expressed in parentheses.

In order to generate the most significant pharmacophore model, the current study proceeds employing the ligand-based three dimensional quantitative structure-activity relationship (3D-QSAR) pharmacophore model constructions. Additionally, these approaches disclose the common important features imbibed within different known chemical compounds essential for retrieving the novel compounds from the databases. For the present study, 30 training set compounds, Figure 1, were utilized to build the qualitative hypothesis using *HypoGen* algorithm. The activity values of the 30 training set compounds spanned between 0.1~470,000 nmol/L. As a result, a total of ten hypothesis were successfully generated utilizing the chemical features present within the training set compounds combined with the statistical parameters that include cost valve, correlation (r), fit values and RMSD as depicted in Table 1.

Introspecting the ten hypotheses, it was revealed that the six hypothesis namely Hypo 1, Hypo 2, Hypo 3, Hypo 4, Hypo 5 and Hypo 10 expressed the Hydrogen bond acceptor lipid (HBL) and hydrophobic (HyP) features illuminating their importance in cysteine protease inhibition. A marginal variation was observed with Hypo 6, Hypo 7, Hypo 8 and Hypo 9 which represented the HyP and 3 ring aromatic (RA) features. Furthermore, the RA feature has been demonstrated by all the models implying that it might be a key feature in rendering biological activity, Table 1.

**Figure 2:**
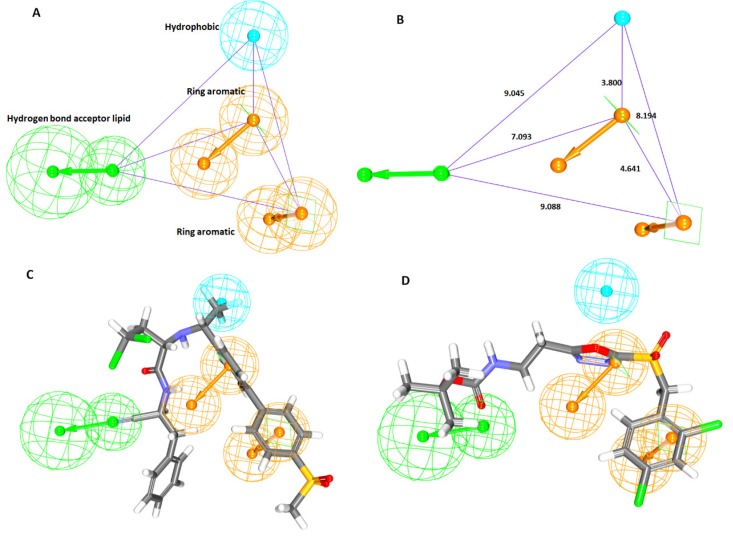
Generated pharmacophore, Hypo 1 with its features and geometry. (**A**) Demonstrates the best pharmacophore model with its features, 1HBL, 1HyP and 2RA. (**B**) Corresponds to the geometry of four features. (**C**) Represents the mapping of best pharmacophore model Hypo1 to the most active compound (IC_50_ 0.1 nmol/L) in the training set. The compound was seen to align with four features 1HBL, 1HyP and 2RA. (**D**) Refers to the most inactive compound (IC_50_ 470,000 nmol/L) from the training set. The compound was found to align with only three features, 1HBL and 2RA.

Correspondingly, an ideal pharmacophore model was chosen based upon the Debnath’s analysis that is governed by the highest cost difference, low total cost, high correlation complemented by low RMSD value and greater fit values. Cost difference refers to the difference between the null and the total cost hypothesis. Correspondingly, a difference higher than 60 bits, the hypothesis corresponds to have a correlation probability approximately ≥90%, while the bit difference between 40–60 implies that the hypothesis has a correlation probability ranging between 70~90% [35]. Furthermore, the correlation coefficient is a geometric index and relies on the linear regression and the RMSD values corresponds to the deviation between the experimental values and the predicted activity values and therefore, the lower RMSD values are to be considered. Since Hypo 1 obeys to all the Debnath’s parameters, it was selected as an ideal pharmacophore to retrieve the potential leads from databases upon validation. The corresponding Hypo 1 rendered low total cost of 214.15, high cost difference of 66.39, low RMSD of 2.42 and high correlation of 0.71, displaying two RA, one HBL and one HyP features complemented by highest fit value, Table 1. The pharmacophore model with four features, Hypo 1 was built, Figure 2A and its geometrical constraints are shown in Figure 2B. Fit values significantly elucidate the chemical meaning of the pharmacophore and govern to overlap the molecules onto the pharmacophore. The most active compound in the training set has demonstrated a fit value of 7.98 while the least active compound has rendered a fit value of 4.14. Correspondingly, the most active compound with the an IC_50_ value of 0.1 nmol/L has aligned to all the pharmacophore features, Figure 2C and least active compound bearing IC_50_ value of 470,000 nmol/L has displayed only three chemical features, Figure 2D.

#### 2.1.2. Receptor-Based Pharmacophore Model Generation (Pharm 2)

Structure-based pharmacophore logically illustrates the key residues responsible for the binding pattern of the ligand and the protein. In the current study, this method was exploited considering the Cruzain protein 1ME3 which is in complex with hydroxymethyl ketone inhibitor (II) enabling the *Receptor-Ligand Pharmacophore Generation* protocol embedded with the DS. Correspondingly, 10 pharmacophore models have been generated with varied number of features, types of features and the selectivity score as tabulate in Table 2.

**Figure 3:**
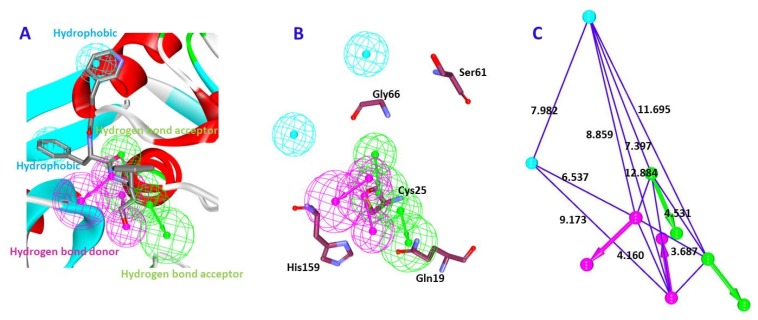
Structure-based pharmacophore generation. (**A**) Pharmacophore model displaying different features against the bound inhibitor. (**B**) Pharm 2 representing key residues. (**C**) Interfeature distance displayed by pharm 2.

Amongst the generated pharmacophore models, the pharmacophore model1 with six features (hereinafter pharm 2) was chosen based upon the highest selectivity score, Table 2 and Figure 3A. Contemplating on the features, it can be elucidated that the key residues were represented by the pharmacophore features as depicted in Figure 3B. Accordingly, pharm 2 has represented 2 hydrogen bond acceptor (HBA), 2 hydrogen bond donor (HBD) and 2 Hydrophobic (HyP) features, Figure 3A. Their corresponding interfeature distance was displayed in Figure 3C. Furthermore, all the models were imbibed with hydrophobic features and two hydrogen bond acceptor features.

### 2.2. Validation of the Pharmacophore Model

To assess the robustness of the generated pharmacophore model in retrieving the lead-like compounds from the database, validation was performed executed by Fischer’s randomization method for pharm 1 and ROC for pharm 2. Additionally, a common validation was executed for both the models employing the decoy set method.

**Figure 4:**
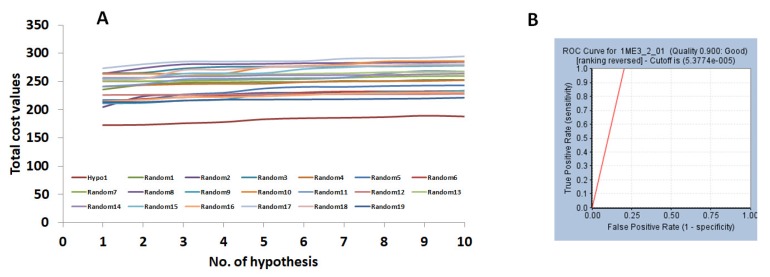
Validation of the pharmacophore models. (**A**) Evaluates the difference in cost between 19 scrambled runs selecting 95% confidence level. Pharm 1 had displayed a lower cost value. (**B**) Depicts the ROC curve of pharm 2 projecting the model to be of good quality.

The Fischer’s randomization method was applied to assess the quality of the hypothesis and judge if the model was generated arbitrarily. This was performed together with the generation of the hypothesis using 95% confidence level and the significance was computed as described previously. Consequently, 19 random spreadsheets were obtained that displayed a higher cost values than the Hypo 1 (pharm 1), stating that the Hypo 1 was not generated by chance and affirms the superior quality of the model, Figure 4A. 

The pharm 2 was validated by the Receiver Operating Characteristic (ROC) alongside the generation of the pharm 2 with 15 actives and 20 inactives. Pharm 2 has identified 17 compounds as actives with two false positives. Correspondingly, the area under curve was observed to be close to 1 with the quality of the pharmacophore being a good model, Figure 4B.

Furthermore, the decoy set method of validation was applied by instituting an external dataset with 1000 compounds composed with 25 active compounds. The pharm 1 has retrieved a total of 27 compounds (Ht) containing 22 actives (Ha), while pharm 2 has mapped to 31 compounds (Ht) with 25 active compounds (Ha). The GH score was computed to be 0.80 for pharm 1 and 0.73 for pharm 2, respectively, thereby manifesting the significance of both the models in identifying the active potential compounds, Table 3.

### 2.3. Virtual Screening for Redeeming the Lead-Like Candidates

**Figure 5:**
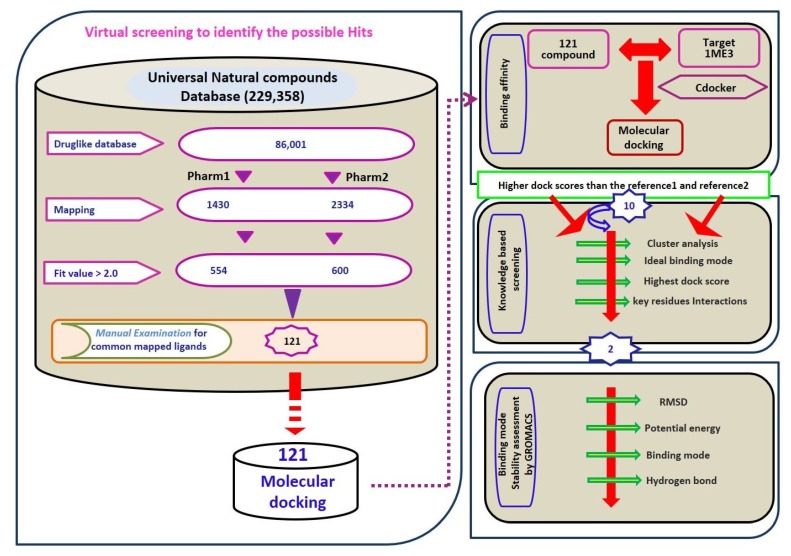
Overview of the screening methodology adapted.

Virtual screening is a sequential process of retrieving the prospective drug candidates upon applying certain filters using chemical features imbibed on both the pharmacophore models as the 3D query. Universal Natural Products Database (UNPD) (http://pkuxxj.pku.edu.cn/UNPD) [36] was utilized for recognizing the lead candidates which consisted of 229,358 molecules. Correspondingly, the database was subjected to drug-like assessment using the Ro5 and ADMET approaches that resulted in 86,001 compounds. Upon subjecting them to ligand pharmacophore mapping, pharm 1 has mapped to a total of 1430 compounds and pharm 2 has retrieved 2334 compounds. The compounds were further sorted based upon the fit value greater than 2 filtering 554 compounds and 600 compounds respectively. The obtained ligands were manually probed for retrieving the compounds representing the features of pharm 1 and pharm 2. Consequently, the obtained 121 compounds were subjected to molecular docking mechanism employing Cdocker accessible on the DS. The sequential process of screening has been illustrated in Figure 5. 

### 2.4. Molecular Docking Studies

Molecular docking is a mechanism which delineates on the degree of binding affinities that exist between the protein and the inhibitors demonstrated by -Cdocker interaction energies. The active site residues portray a crucial role in imparting the inhibitory activity. From the knowledge garnered from the crystal structure bearing the PDB code 1ME3, the active site residues were identified as Gln19, Cys25, Ser61, Gly66, Asp158 and His159. The compounds obtained from the above steps along with the most active compound from the training set were escalated to docking protocol. Hereinafter, the most active compound is labelled as reference 1 and the co-crystallized ligand is referred to as reference 2 that were employed to evaluate the retrieved compounds. From the largest cluster generated by the docked poses, the best binding pose was elected considering the highest dock score than the reference and the interactions with the key residues. The reference compounds have generated a -Cdocker interaction energy of 47.79 and 50.69, respectively. All those compounds that expressed higher score than the reference were considered as potential Hits [37] (active drug-like candidate compounds) which resulted in 10, compounds Supplementary Figure S1, which were scrupulously examined for the interactions with active residues located at the proteins binding pocket. As a result, two compounds have been obtained that showed higher -Cdocker interaction energies of 53.57 and 51.84, respectively, corroborated by key residue interactions. Therefore, these compounds were regarded as Hit1 and Hit2 and were thoroughly studied using MD simulation executed using GROMACS.

### 2.5. Molecular Dynamics Simulations

**Figure 6:**
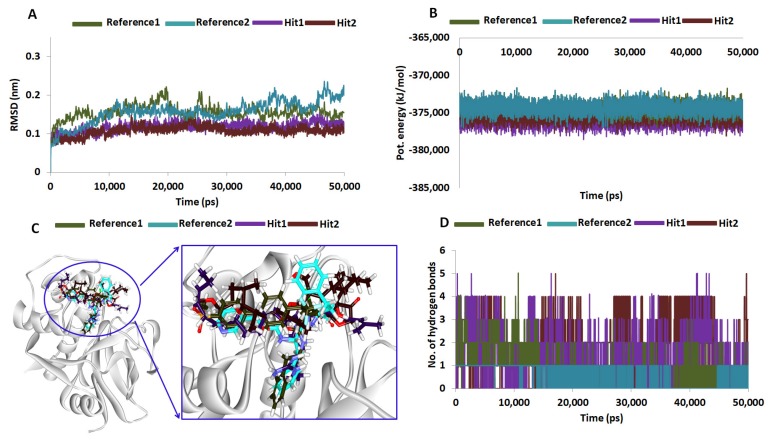
Graphical representation of the molecular dynamics simulation studies conducted during 50 ns. (**A**) Represents the RMSD profiles for the backbone atoms of four systems. (**B**) Demonstrates the potential energies plotted for each system. Both the figures demonstrate that the systems were well converged. (**C**) Binding pattern of reference and the Hits within the proteins binding pocket. The Hits and reference have displayed a similar binding pattern. (**D**) Number of intermolecular hydrogen bonds between protein and Hits during 50 ns simulation. The Hits have displayed a higher number of hydrogen bonds.

To decipher on the molecular docking results and to further elucidate on the binding stability of the chosen Hit compounds, they were subjected to MD simulation along with the reference compounds. The docked poses with protein complex were used as the initial MD structures conducted for 50 ns and the findings were evaluated based upon the RMSD and the potential energy profiles. RMSD was computed for the protein backbone atoms and existed between 0.10~0.25 nm. However, it was interesting to note that the RMSD of the Hits were relatively compact at 0.1~0.15 nm while the reference compounds have expressed a higher value, Figure 6A. Additionally, the average RMSD values were computed to be 0.22 nm for reference 1 and 0.19 nm for reference 2, while it was observed to be 0.11 nm and 0.10 nm for Hit1 and Hit2, respectively. These results demonstrated that the protein – Hit complexes were more stable than the reference complex. Additionally, the potential energy profiles also suggest the same and exists between −373,000 ~ −377,000 kJ/mol, Figure 6B. Both the results emphasize the systems were stable throughout 50 ns simulation run. The binding mode assessment of the systems was conducted retrieving the representative structures from the last 5 ns. Upon subsequent superimposition of the representative structures, it was revealed that the Hits have occupied the binding pocket in the similar mode as the reference and were found to anchor with the key residues, Figure 6C.

**Figure 7:**
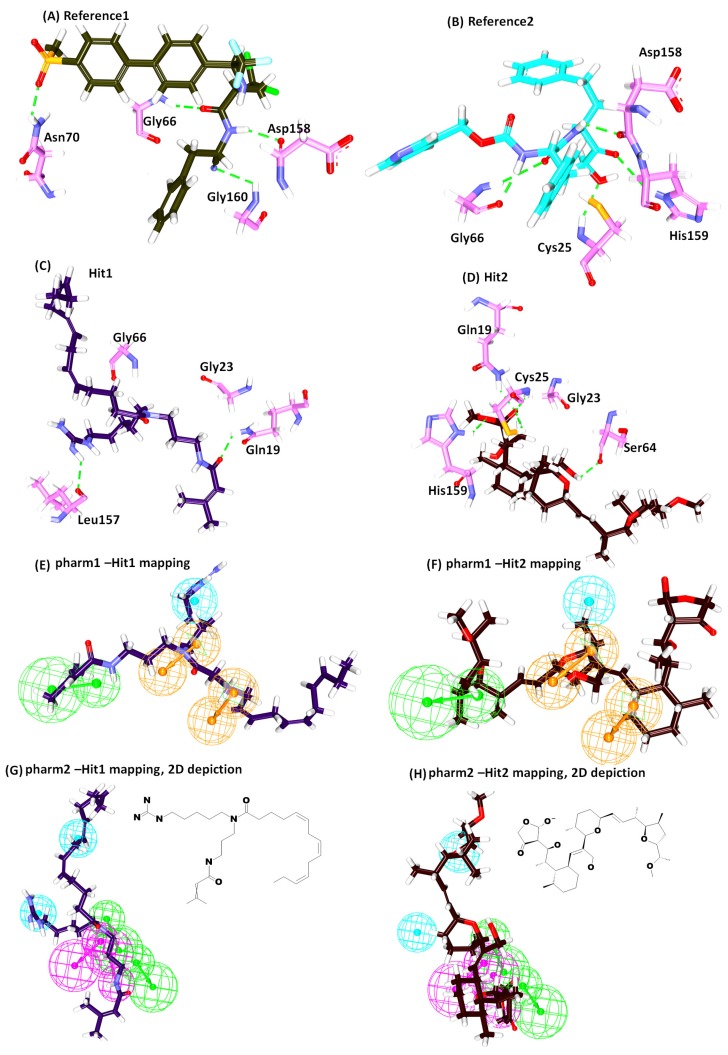
Interactions and the binding pattern of the reference and the Hits. Green dashed lines represent the hydrogen bond interaction (**A**) Hydrogen bond interactions between the protein-reference1. (**B**) Hydrogen bond interactions between the protein-reference2. (**C**) Intermolecular hydrogen bond interactions between the protein-Hit1. (**D**) Intermolecular hydrogen bond interactions between the protein-Hit2. (**E**) Hit1-pharm 1 mapping. (**F**) Hit2-pharm 1 mapping. (**G**) Hit1-pharm 2 mapping and 2D representation of Hit1. (**H**) Hit2-pharm 2 mapping and 2D representation of Hit2.

Interrogating the interactions, it was revealed that reference 1 has generated four hydrogen bonds involving the residues Gly66, Asn70, Asp158 and Gly160, respectively, rendered by an acceptable bond length, Table 4. Furthermore, the residues Leu67 and Ala133 have demonstrated the π-alkyl hydrophobic interactions. The benzene ring A and ring B of the ligand have interacted with the Leu67 with a distance of 4.7 Å and 5.4 Å, respectively. Additionally, ring B was also observed to display a π-alkyl hydrophobic interactions with Ala133 residue rendered by a distance of 3.7 Å. Furthermore, the residues Gln19, Gly23, Trp26, Cys25, Thr59, Ser61, Ser64, Asp60, Gly65, Met68, Asn69, Leu157, His159 and Glu205 assisted in clamping the compounds at the active site of the protein, as depicted in Table 4 and Figure 7A.

Reference compound 2 has represented five hydrogen bonds involving five residues, Cys25, Gly66, Asp158 and His159 with a bond distance below 3 Å, Table 4 and Figure 7B. The residues Asp60, Leu67 and Ala133 have resulted in the electrostatic and hydrophobic interactions, respectively. The residue Asp60 generated an electrostatic π-anion interaction with the benzene ring A of the ligand represented by a bond distance of 4.1 Å. The ring B has interacted with the residues Leu67 and Ala133 with a bond length of 4.7 Å and 3.8 Å, correspondingly, forming a hydrophobic π- alkyl interaction. The residues Gln19, Gly23, Ser24, Trp26, Ser61, Ser64, Met68, Asn70, Leu157, Gly160 and Glu205 have held the ligand by van der Waals interactions thereby locking the ligand firmly at the active site of the protein, Table 4 and Figure 7B. 

Hit1 has formed two hydrogen bonds with residues Gln19 and Leu157 complemented by an agreeing bond length. Additionally, the residues Ala136, Trp177 and His159 have anchored to the protein through the alkyl/ π-alkyl bonds. The atom C26 and C27 of the ligand has joined with Ala136 by the hydrophobic alkyl bond with a bond length of 4.2 Å and 4.4 Å. The benzene ring of charged residue His159 has interacted with C27 by the π-alkyl bond by a bond length 4.7 Å. Besides, another π-alkyl bond was formed with the C26 atom and the benzene ring of the Trp177 with a length of 5.0 Å. Furthermore, Hit1 was locked into the active site by van der Waals interaction involving the residues Cys25, Trp26, Thr59, Asp60, Ser61, Ser64, Gly65, Leu67, Met68, Asn70, Ala133, Val134, Ser139, Asp158 and Gly160, respectively, Table 4 and Figure 7C. 

Hit2 on the other hand has generated five hydrogen bonds with residues Gln19, Cys25, Ser64 and His159 with an acceptable bond distance. The residues, Cys25, Trp26, Leu67 have strongly held the ligand with alkyl/ π- alkyl bonds. The residue Cys25 has interacted with C39 atom of the ligand with alkyl hydrophobic bond rendered by a length of 4.3 Å. A π-alkyl bond was noticed between the benzene ring of Trp26 and the C39 atom of the ligand conferred by a bond length of 5.4 Å. The Leu67 formed alkyl bond with the C42 atom of the ligand rendered by a bond length of 4.7 Å. The residues Gly23, Ser24, Thr59, Asn60, Ser61, Cys63, Gly65, Gly66, Asn70, Asp158, Gly160 and Trp177 have additionally favored proper seating of the ligand into the proteins active site, Table 4 and Figure 7D. These compounds have mapped ideally to all the features of the pharmacophore implying that they are imbibed with the key features needed for the inhibitory activities, Figure 7E–H and their 2D structures are depicted in Figure 7G,H. Moreover, the significance of the Hits was illuminated by the number of hydrogen bonds rendered by them. The reference compounds have generated an average of 0.8 and 0.10 hydrogen bonds while Hit1 delivered 1.4 and Hit 2 was conferred 1.6 hydrogen bonds Figure 6D. Exhaustive details of the interactions are given in Table 4 and Supplementary Figure S2. These findings vividly demonstrate the eminence of the Hits over the reference in manifesting their therapeutic ability.

### 2.6. Binding Free Energy Calculations

**Figure 8:**
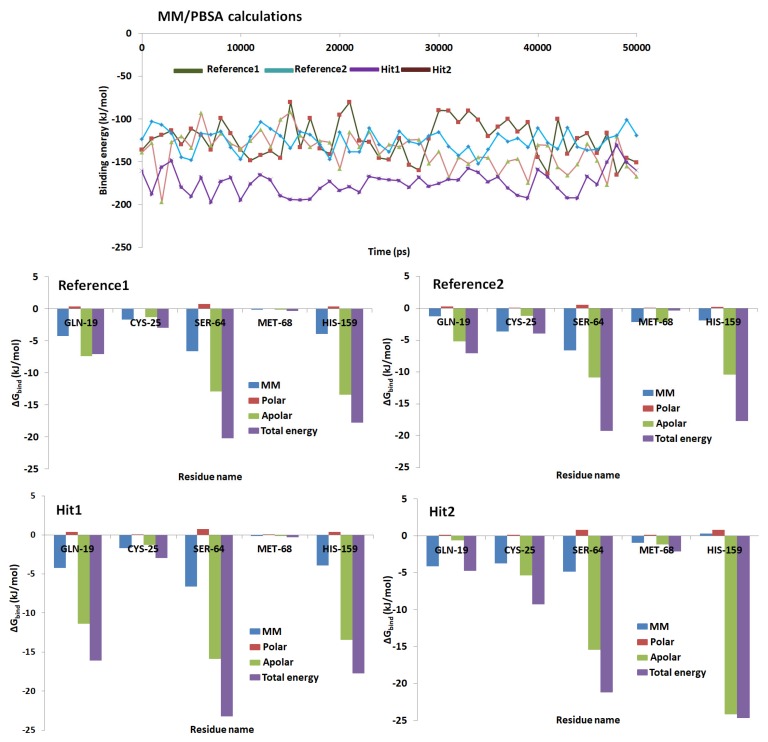
MM/PBSA binding free energy calculations of the Hits and reference compound along with the per-residue contributions.

MM/PBSA calculations were initiated to delineate on the nature of the small molecule and its mode of accommodation at the proteins active site. For its accomplishment, 50 snapshots were evenly extracted and were subjected to MM/PBSA calculations and were elucidated by different energetic components. The reference compounds and the two retrieved Hits have demonstrated the binding free energies between −145 kJ/mol~−120 kJ/mol, Figure 8. The average binding energy for the protein-reference 1, protein-reference 2, protein-Hit1 and protein-Hit2 was calculated to be −123.25 kJ/mol, −125.63 kJ/mol, −172.93 kJ/mol and −133.60 kJ/mol, correspondingly. To gain further insight into the per residue contribution of the key residues, the study was extended to per residue energy contribution available with MM/PBSA. The per residue contributions have shown that the His159 was the largest contributor towards the total energy. The residue Cys25 demonstrated highest contribution in Hits than in the reference compounds. The residue Gln19 contributed largely in Hit 1, while it has showed a respectable contribution in reference compounds and Hit2. Similar pattern noticed with Ser64. The key residue Met68 favored towards the Hits2 than the reference compounds and Hit 1. The details of the per-residue contributions of the key residues were depicted in Figure 8. Furthermore, it could be noticed from Figure 8 that each key residue contribution towards the binding free energy was higher with the Hits upon comparison to the reference compound.

## 3. Discussion

*Trypanosome cruzi* is the causative organism for Chagas disease and is the prime reason for heart diseases in South America. The currently offered treatments are reported to be associated with toxicity or have shown to develop resistance towards the disease and hence hamper effective treatment. These direct towards the dire necessity for designing and discovering new drugs.

In order to develop new drugs with low toxicity, an exhaustive dual pharmacophore virtual screening was performed to redeem potential lead like candidate compounds imbibed with a potential cure to Chagas. A systematic filtering techniques followed by the molecular docking and the molecular dynamics simulations have portrayed two compounds as potential antichagastic agents executed employing Cdocker accessible on the DS and GROMACS. Therefore, in order to discover the most efficient ligands, the criteria of selection includes, ligands from maximum clustered group displaying the dock score higher than the reference compounds, interactions with key residues, ideal binding mode, ligand occupation of S1, S2 and S3 subsites were considered. The resultant findings have demonstrated that the identified Hits have been locked in the active site interacting with the key residues and various charged amino acids.

The active site of Cruzain is represented by seven subsites observed binding to the peptide subunit [3] with four subsites on the acyl side and three subsites on the amino side. The subsites on the acyl side, S4, S3, S2 and S1 bind to peptide amino acids P4, P3, P2 and P1 while the amino subsites S1’, S2’ and S3’ interact with the peptide amino acid P1’, P2’ and P3’ [3]. Amongst these subsites, S1, S2 and S1’ are considered to be well defined with the subsites S2 and S1 induce specificity [34,38]. Additionally, it is documented that the residues Gly66 and Leu67 are crucial in forming the hydrogen bond interactions and harboring hydrophobic groups located at the S2 subsite and thus promote the shape complementarity of small molecules at the cleft [11]. Accordingly, the ligands in close proximity to these residues were additionally considered to retrieve the candidate compounds.

**Figure 9:**
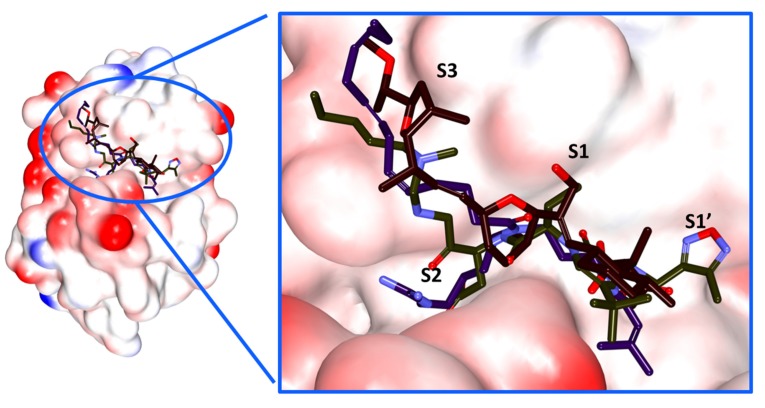
Pictorial illustration of the accommodation of the ligands in the proteins subsites.

We attempted to understand the binding mode of the identified Hits and their accommodation within the subsites. Hit1 has occupied the active site extending between S3, S1, S2 and S1’ subsites by forming hydrogen bonds and the hydrophobic interactions. The Gln19 and Leu157 residues belonging to the subsites near to S1 and S2 have favored the formation of the hydrogen bond interactions. Gly66 from the S2, S3 subsite resulted in the formation of weak hydrogen bond rendered by a bond length of 3.3 Å. The residue that lie near to the hydrophobic surface of S1’ sub unit, Ala136 has anchored with C26 and C27 of the ligand by an alkyl bond with a bond lengths of 4.2 Å and 4.4 Å, respectively. Yet another residue Trp177 in close vicinity to S1’ sub unit, has joined to the C26 atom of the ligand with its benzene ring resulting in the π-alkyl bond displaying a bond length of 5.0 Å. The other residues such as Gly23, Cys25, Trp26,Thr50, Asp60, Ser61, Ser64, Gly65, Gly66, Leu67, Met68, Asn70, Ala133, Val134, Ser139, Asp158 and Gly160 of different subsites have participated in the van der Waals interactions, Figure 9.

Hit2 was found to be locked within the active sites through the residues employing different interactions. The key catalytic residues, Cys25 and His159 have participated through the hydrogen bond interactions. Gln19, a residue that lies in proximity to S1 subsite rendered hydrogen bond interaction as seen with the Hit1. Additionally, the Ser64 from the vicinity of S3 subsite has involved in the hydrogen bond interactions. It is interesting to note that the residues at 61, 64 and 67 are non-conserved residues imparting selectivity [34]. Moreover, Cys25 has formed hydrophobic alkyl bond with the C39 atom of the ligand with a bond length of 4.3 Å. The indole ring of Trp26 that maybe from S2 subsite has formed the π-alkyl bond demonstrated by a length of 5.4 Å. Additionally, Leu67 from the vicinity of S3 subsite formed an alkyl bond with the C42 atom of the ligand demonstrated by a bond distance of 4.7 Å, Figure 9. The residues, Gly23, Ser24, Thr59, Asn60, Ser61, Cys63, Gly65, Asn70, Asp158, Gly160 and Trp177 from different subsites have participated in the van der Waals interactions. It can therefore be understood that the Hits were firmly positioned at the active site forming various bonds from different sub-sites. Furthermore, focusing on the interactions, it was noticed that the residues Gly23, Gly65, Asn70 and Gly160 have appeared to from various interactions including the van der Waals interactions with the Hits, Supplementary Figure S2. The residues Asn70 and Gly23 hold the inhibitors at each ends while they are held firmly at the center by Gly65 and Gly160, thereby locking the inhibitors tightly at the active site apparently seeming like a *tetrad* mode of inhibition, Supplementary Figure S4. This guides us to speculate that the interactions with the tetrad residues might impart effective therapeutics.

Additionally, the Hits have demonstrated hydrogen bond interaction with the key residues as described in the previous literature [3,4,11,39]. The Hits have recorded higher molecular dock scores and lower binding energies than the reference compounds complemented by higher number of hydrogen bonds and stable RMSD and potential energy profiles. These findings suggest that the identified Hits can serve as potential lead candidates against Chagas disease.

## 4. Materials and Methods

### 4.1. Preparation of the Dataset

A dataset consisting of 30 compounds were carefully instituted that have reported different inhibitory activity (IC_50_) values against Cruzipain of *T. cruzi* [40]. These compounds have displayed varied structures and IC_50_ values ranged between 0.1 nmol/L~470,000 nmol/L and were employed to build the hypothesis. Specifically, the training set should consist of the most active compounds, a minimum of 16 structurally diverse compounds that can exhibit 4–5 order magnitude and to refrain the addition of known inactive compounds due to steric hindrance [35].

### 4.2. Generation of the Pharmacophore Model

#### 4.2.1. Ligand-Based Pharmacophore Model Generation (Pharm 1)

Employing 30 compounds, different qualitative hypotheses were constructed utilizing the HypoGen *3D QSAR pharmacophore generation protocol* implemented in the DS [41,42]. The *best* algorithm was chosen to generate the low energy conformation for each compound at 20 kcal/mol as the cut-off. Unlike *fast* algorithm, *best* algorithm tweaks the bond distances, their angles and the dihedral angles of the generated conformers in order to obtain the superlative model. Further, the uncertainty value has an eminent role in the model generation and thus influences the division of the compounds present in the dataset as active and inactive compounds and is subsequently employed during the subtractive and the construction phases. This value is defined as the ratio of the reported activity value relative to the minimum and the maximum values and must be higher than 1.0 [35]. Accordingly, for the current investigation, a default value of 3.0 has been retained.

To gain knowledge on the important features imbibed by the training set compounds, the *feature mapping* module present on the DS was initiated which computes about 1000 features existing within the compounds and serves as the key inputs for the model construction. In this pursuit, the *HypoGen* algorithm evaluates the activity of the compounds present in the training set and a minimum of 0 and a maximum of 5 features were selected. From the generated 10 hypothesis, the best hypothesis was chosen based on Debnath’s analysis.

#### 4.2.2. Receptor-Based Pharmacophore Model Generation (Pharm 2)

To retrieve the most potential inhibitor that abides by the key inhibitory features, a receptor-based pharmacophore model was generated considering all the available crystal structures for Cruzain. Upon corresponding super imposition of the available structures using *Align Structures* protocol available with the DS, it was perceived that the key residues were conserved in all the structures (data not shown). Accordingly, in the current study, to generate a structure-based pharmacophore model, the structure of Cruzain bound (PDB code: 1ME3) to a hydroxymethyl ketone inhibitor was chosen. The binding pattern of the ligand was critically analyzed and revealed that the residues Gly19, Cys25, Ser61, Gly66 and His159 were crucial in holding the ligand at the binding site. Correspondingly, *Interaction Generation* module present with the DS was enabled to label the residues in close proximity around 8.0 Å of the ligand to prompt the pharmacophore features. Subsequently, the Receptor-ligand Pharmacophore Generation protocol was utilized to generate the pharmacophore models with fast/flexible options.

### 4.3. Validation of the Generated Pharmacophore

The ligand-based pharmacophore (pharm 1) was validated using Fischer’s randomization method in order to ensure the robustness of the pharmacophore in efficiently retrieving the lead candidates from the databases. The Fischer’s randomization was performed along with the generation of the pharmacophore model at significance level of 95% [35]. The test is executed alongside the generation of the original hypothesis by jumbling the activity values present in the training set at specific significance confidence of 90%, 95%, 98% and 99% [35]. Correspondingly, 19 random spreadsheets were generated by jumbling the training set activity values. The structure-based pharmacophore (pharm 2) was validated by Receiver Operating Characteristic (ROC method) taking 15 active and 20 inactive compounds. The obtained results were read according to the Area Under Curve (AUC) with the computed values representing between 0 to 1. The scale of the AUC were as follows 0.90–1.00 is excellent, 0.80–0.90 being good, 0.70–0.80 stated as fair, 0.60–0.70 being poor and 0.50–0.60 is considered fail.

Furthermore, a common validation test was conducted for both the models using the decoy set method, instituting an external dataset of 1000 compounds consisting of 25 actives. The two pharmacophore models were subjected to map against the compounds in the database by enabling the *Ligand Pharmacophore Mapping* equipped with the DS. The obtained results were examined based upon the formula.

$$EF=\frac{Ha\;X\;D}{Ht\;X\;A}$$

$$GF=\left(\frac{Ha}{4HtA}\right)\left(3A+Ht\right)X\;\left\{1-\frac{Ht-Ha}{D-A}\right\}$$

### 4.4. Virtual Database Screening and Drug Like Assessment

The validated pharmacophore was subsequently allowed to screen the databases to claim the chemical compounds with the features characteristic to the pharmacophore model. Logically, the retrieved molecules are presumed to be potential candidates against the Chagas disease. Correspondingly, the *Ligand Pharmacophore Mapping* protocol available on the DS was initiated with *rigid* fitting method and the *best* mapping was opted as *true*. Subsequently, a large database, universal natural compound (UNP) database was subjected to sieving and the compounds that map with the features of the pharmacophore were obtained.

Drug likeness assessment was performed to evaluate the pharmacokinetic properties of a drug. A compound with low pharmacokinetic properties may result in declining from its further development. In order to ensure the pharmacokinetic properties of the retrieved compounds, the absorption, distribution, metabolism, excretion and toxicity (ADMET) and the Lipinski’s rule of five (Ro5) was applied. Specifically, ADMET assesses if the drug is able to cross the blood-brain barrier (BBB) with suitable human intestinal absorption (HIA/absorption) and the solubility. For the present investigation, an upper limit for BBB, HIA and solubility were selected as 3, 0 and 3 respectively [43,44]. Following this, the obtained compounds were subjected to Ro5 which determines a chemical compound to be orally active and thus labels a drug to be pharmacological or biologically active.

### 4.5. Molecular Docking Studies

In order to assess the affinity between the protein and the ligand molecular docking was performed that computes a precise score for each ligand and correspondingly infers on the ligand that best fits within proteins active site. Additionally, the molecular docking gives an insight on the behavior of the ligand at the active site and further determines the ideal binding mode referred to as *pose*. Logically, molecular docking elucidates the ligand and its corresponding key residue interactions at the atomic level. For the current study, Cdocker, available on the DS was employed that operates using CHARMm wherein the protein is held rigid while the ligands are allowed to move and further facilitates the placement of the ligands within the defined binding site sphere. The results are evaluated based upon -Cdocker interaction energies, higher the -Cdocker interaction energies greater is the binding affinity between the protein and the ligand [44].

For the current investigation, the drug target, cysteine protease (PDB code: 1ME3) [39] was retrieved from protein data bank (https://www.rcsb.org/) with a superior resolution of 1.2 Å, bearing a co-crystallized ligand. The chosen protein was subsequently prepared by discarding all the water molecules and supplementing with the hydrogen atoms by applying the CHARMm force field. Following its preparation, the active site of the protein was determined at 10 Å around the cocrystal by considering the volume of the complexed compound. Correspondingly, all the residues that lie within 10 Å were referred to as key residues. To further authenticate the docking protocol, the cocrystal was docked into the active site of the protein, which resulted in the generation of the docked pose with an acceptable root mean square deviation (RMSD) of 1.3 Å, Supplementary Figure S3. Therefore, these parameters were chosen to conduct the molecular docking with the obtained ligands and each ligand was permitted to generate 60 poses. The best binding mode and the potential ligands were determined from the maximum cluster, highest dock scores than the reference and interactions with the key active residues. The redeemed ligands were subjected to molecular dynamics (MD) simulation studies to assess the stability of the complex system and to further affirm the docking results.

### 4.6. Molecular Dynamics Simulation Studies

Molecular dynamics studies were widely applied to delineate on the molecular movement, predict the enzyme mechanism and further to comprehend on the complex assemblies. MD simulations additionally impart knowledge on the behavior of the small molecules with its protein counterpart at the atomistic level [45,46,47]. For the current investigation, MD was employed to assess the stability of the target and the ligand complexes and were studied in terms of RMSD and the potential energies recruiting GROningen MAchine for Chemical Simulations (GROMACS v5.0.6). The identified ideal binding modes of the cysteine protease in complex with the retrieved inhibitors from the molecular docking results were used as the initial structures for MD recruiting all atom CHARMm27 force field [48]. Subsequently, the ligand topologies were generated employing SwissParam [49]. A dodecahedron water box [43] was generated to execute the simulation and was solvated with TIP3P water model and was neutralized with counter ions. The energy minimization was conducted using the steepest descent algorithm that facilities the relaxation of the initial structures by evading rigid hindrances. Additionally, the number of steps were confined to 10,000 using the minimization force less than 10,000 kJ/mol. Following this, a double stepped equilibration was conducted by NVT and NPT, respectively. The first step of equilibration was executed with a constant number of particles, volume and temperature complex (NVT) for 1 ns at 300 monitored by V-rescale thermostat. The second equilibration step was performed for number of particles, pressure and temperature (NPT) ensemble for 1 ns controlling the pressure at 1 bar with Parrinello-Rahman barostat [50]. During the equilibration steps the backbone of the protein was refrained, while permitting the movement of the solvent molecules and the counter ions. The equilibrated ensembles were subjected to MD simulations conducted for 50 ns employing LINCS and SETTLE [51,52] algorithm for bond constraints and geometry of water molecules. Particle Mesh Ewald (PME) [53] method was used to compute and calculate the long-range electrostatic interactions and defining a cut-off value of 9 Å and 14 Å for short-range interactions and van der Waals interactions, correspondingly. The obtained results were evaluated employing visual molecular dynamics (VMD) [54], GROMACS and DS.

### 4.7. Binding Free Energy Calculation

The binding free energies for each system were conducted to evaluate any conformational changes employing the Molecular Mechanics/Poisson–Boltzmann Surface Area (MM/PBSA) compiled for GROMACS v5.0. 50 snapshots were evenly extracted from last 5 ns trajectories for the execution of the MM/PBSA. The computed ΔG considers both the protein and ligand fluctuations thereby ensuring a conventional positioning of the ligand molecule.

The binding free energy protein-ligand complex in solvent system can be defined as

ΔG_binding_ = G_complex_ − (G_protein_ + G_ligand_) (1)

Herein, G_complex_ indicates the total free energy of the complex and G_protein_ and G_ligand_ denotes the separated protein and ligand in the solvent. Their corresponding free energies can be computed by

G_X_ = E_MM_ + G_solvation_ (2)

where, X represents a protein, ligand or its complex. E_MM_ refers to the average molecular mechanics potential energy in vacuum, while the G_solvation_ implies to the free energy present in the solvation.

Furthermore, the molecular mechanics potential energy in vacuum can be calculated by using the equation

E_MM_ = E_bonded_ + E_non-bonded_ = E_bonded_ + (E_vdw_ + E_elec_) (3)

E_bonded_ refers to the bonded interactions, while the non-bonded interactions are expressed by E_non-bonded_. ΔE_bonded_ is generally regarded as zero [51,55].

The solvation free energy (G_solvation_) is denoted by the sum of electrostatic solvation free energy (G_polar_) and apolar solvation free energy (G_non-polar_) and is given as following

G_solvation_ = G_polar_ + G_non-polar_ (4)

G_polar_ is computed recruiting the Poisson-Boltzmann (PB) equation [56] while G_non-polar_ is computed from the solvent-accessible surface area (SASA) and can be written as below

G_non-polar_ = γSASA + b (5)

Here, the γ represents the coefficient of the surface tension of the solvent and b is its fitting parameter, with values 0.02267 kJ/mol/Å^2^ or 0.0054 kcal/mol/Å^2^ and 3.849 kJ/mol or 0.916 kcal/mol, respectively.

## 5. Conclusions

Chagas disease, also called as American trypanosomiasis, is a neglected tropical disease caused by *T. cruzi* is one of the leading causes of heart diseases [3,57]. Because of the ineffectiveness of the currently available treatments, the research in identifying new drugs has an increasing demand. The present study adapts the 3D-QSAR and structure based pharmacophore search to retrieve the chemical compounds with inhibitory activity. The corresponding coherent evaluation has retrieved the compounds have that obeyed to all the pharmacophore features from the maximum clustered group resulted from the molecular docking. Subsequently, the Hits have shown higher -Cdocker interaction energies, lower binding free energies than the reference compound supplemented by the charged residues holding the candidate leads through van der Waals interactions. Additionally, the protein-Hit complexes were seated in the similar binding mode noticed with the reference occupying the various subsites rendered by stable RMSD and potential energy profiles during 50 ns simulation run. Furthermore, the Hits have demonstrated higher number of hydrogen bonds computed during 50 ns. Upon probing into the similarity search employing the online tools, it was established that the retrieved compounds have not been analyzed against Chagas disease. Taken together, we therefore advocate that the Hits might serve as effective drugs against Chagas diseases or might define as new scaffolds for the development of new drugs.

## Figures and Tables

**Table 1:** Statistical and predictive significance presented in cost values for top 10 hypotheses generated due to 3D QSAR pharmacophore modelling.

Hypo No	Total Cost	Cost Difference ^a^	RMSD	Correlation	Features ^b^	Maximum Fit
Hypo 1	214.15	66.93	2.42	0.71	HBL, HyP, RA, RA	8.24
Hypo 2	216.71	66.82	2.45	0.70	HBL, HyP, RA, RA	7.79
Hypo 3	216.72	63.81	2.45	0.70	HBL, HyP, RA, RA	7.92
Hypo 4	218.92	61.61	2.48	0.66	HBL, HyP, RA, RA	7.23
Hypo 5	219.39	61.14	2.49	0.59	HBL, HyP, RA, RA	7.97
Hypo 6	220.94	59.59	2.51	0.66	HyP, RA, RA, RA	7.69
Hypo 7	221.84	58.70	2.52	0.68	HyP, RA, RA, RA	7.70
Hypo 8	222.14	58.39	2.53	0.68	HyP, RA, RA, RA	7.62
Hypo 9	222.55	57.98	2.53	0.68	HyP, RA, RA, RA	7.01
Hypo 10	224.35	56.16	2.55	0.67	HBL, HyP, RA, RA	7.92

^a^ Cost difference: difference between the null cost and the total cost. The null cost of ten scored hypotheses is 280.54, the fixed cost value is 126.07 and the configuration cost is 24.03. All costs are represented in bit units. ^b^ HBL: hydrogen bond acceptor lipid; RA: ring aromatic; HYP: hydrophobic.

**Table 2:** Different receptor-ligand based pharmacophores and their features.

Pharmacophore Model	Number of Features	Feature Set *	Selectivity Score
Model1	6	HyP, HyP, HBD, HBD, HBA, HBA	11.63
Model2	6	HyP, HBD, HBA, HBA, HBA, HBA	10.72
Model3	6	HyP, HBD, HBA, HBA, HBA, HBA	10.72
Model4	6	HyP, HyP, HBD, HBD, HBA, HBA	10.72
Model5	6	HyP, HyP, HBD, HBD, HBA, HBA	10.72
Model6	6	HyP, HyP, HBD, HBD, HBA, HBA	10.72
Model7	6	HyP, HyP, HBD, HBD, HBA, HBA	10.72
Model8	6	HyP, HyP, HBD, HBD, HBA, HBA	10.72
Model9	5	HyP, HyP, HBD, HBD, HBA	10.12
Model10	5	HyP, HBD, HBD, HBA, HBA	10.12

* HyP: Hydrophobic, HBA: hydrogen bond acceptor, HBD: hydrogen bond donor.

**Table 3:** Different values computed by pharm 1 and pharm 2 employing decoy set method.

Parameters	Pharm 1	Pharm 2
Total number of molecules in database (D)	1000	1000
Total number of actives in database (A)	25	25
Total number of hit molecules from the database (Ht)	27	31
Total number of active molecules in hit list (Ha)	22	24
% Yield of active (Ha/Ht)	81.4	77.4
Enrichment Factor (EF)	32.59	30.96
False negatives (A − Ha)	3	1
False positives (Ht – Ha)	5	7
Goodness of fit score (GF)	0.80	0.73

**Table 4:** Comprehensive interaction of the key residues with the ligands and their dock scores.

Name	-Cdocker Interaction Energy (kcal/mol)	Hydrogen Bond (<3 Å)	Alkyl/π-alkyl	van der Waals Interactions
* Ref1	47.79	Gly66:NH-O23 (2.2) Asn70:HD22-O16 (2.1) Asp158:O-H56(2.0) Gly160:HN-N26 (2.7)	Leu67, Ala133	Gln19, Gly23, Trp26, Cys25, Thr59, Ser61, Ser64, Asp60, Gly65, Met68, Asn69,Leu157, Leu159, Glu205
* Ref2	50.69	Cys25:HN-O3 (2.3) Gly66:HN-O16 (2.8) Gly66:HN-H58 (1.9) Asp158:O-H49 (2.0) His159:HD1-O4 (2.7)	Leu67, Asp60, Ala133	Gln19, Gly23, Ser24, Thr59, Ser61, Ser64, Met68 Asn70, Leu157, Gly160, Glu205,
Hit1	53.57	Gln19:HE22-O20 (2.1) Leu157:O-H74 (2.3)	Ala136, His159, Trp177	Gly23, Cys25, Trp26, Thr50, Asp60, Ser61, Ser64, Gly65, Gly66, Leu67, Met68, Asn70, Ala133, Val134, Ser139, Asp158, Gly160
Hit2	51.84	Gln19:HE22-O21 (2.0) Cys25:HN-O21 (2.0) Cys25:HG-O21 (2.3) Ser64:O-H78 (2.1) His159:HD1-O14 (2.1)	Cys25, Trp26, Leu67	Gly23, Ser24, Thr59, Asn60, Ser61, Cys63, Gly65, Asn70, Asp158, Gly160, Trp177

* Ref implies reference.

## Supplementary Materials

Supplementary figures: Supplementary Figure S1. 2D structures of 8 highest dock scored ligands. Supplementary Figure S2. 2D representation of detailed molecular interaction between the protein and the ligands. Supplementary Figure S3. Aligning of the Co-crystal (pink) overlapped with its docked pose (blue). Supplementary Figure S4. Location of four residues forming a tetrad.
