# Supplementary material for: Discovery of Non-Peptidic Compounds against Chagas Disease Applying Pharmacophore Guided Molecular Modelling Approaches
Source: Molecules. 2018 Nov 22;23(12):3054. doi: 10.3390/molecules23123054 (PMC6321154; doi:10.3390/molecules23123054)
Supplement: Supplementary file 1 [file molecules-23-03054-s001.pdf]

Discovery of Non-Peptidic Compounds against Chagas Disease Applying Pharmacophore Guided Molecular Modelling Approaches

Shailima Rampogu†, Gihwan Lee† , Ayoung Baek, Minky Son, Chanin Park, Amir Zeb, Sang Hwa Yoon, Suhyeon Park and Keun Woo Lee*

[1] **Division of Life Science, Division of Applied Life Science (BK21 Plus), Plant Molecular Biology and Biotechnology Research Center (PMBBRC), Research Institute of Natural Science (RINS), Gyeongsang National University (GNU), 501 Jinju-daero, Jinju 52828, Republic of Korea**

* **Correspondence: kwlee@gnu.ac.kr**

†**These authors contributed equally.**

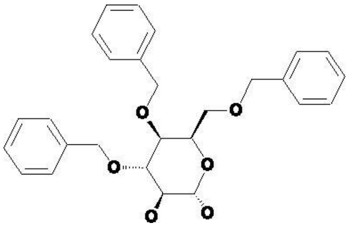

Compound 1

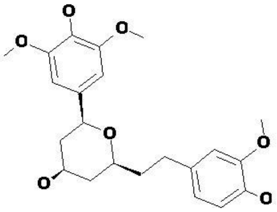

Compound 2

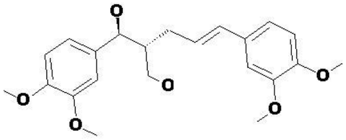

Compound 3

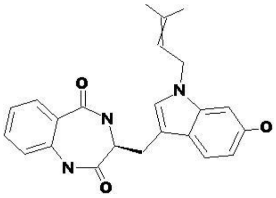

Compound 4

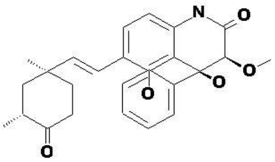

Compound 5

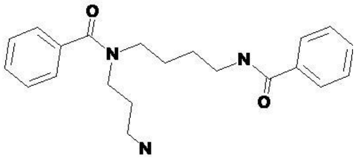

Compound 6

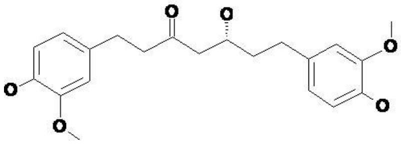

Compound 7

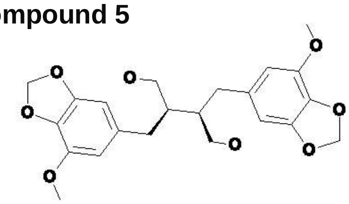

Compound 8

Supplementary figure 1.

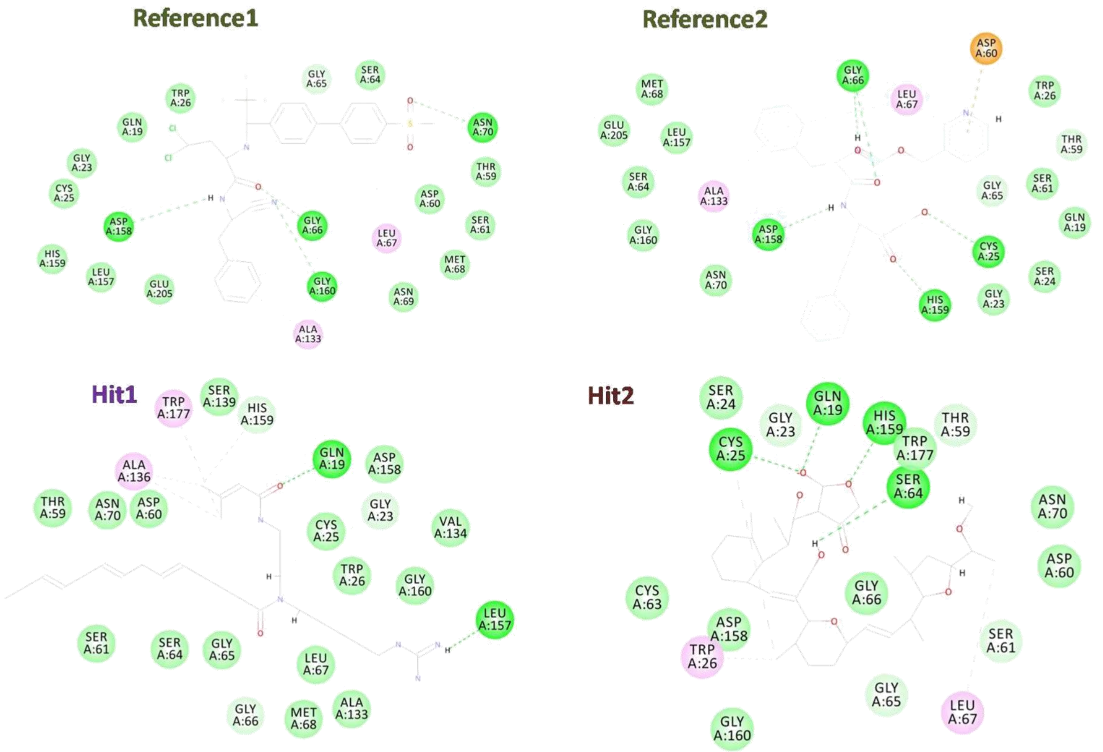

Supplementary figure 2.

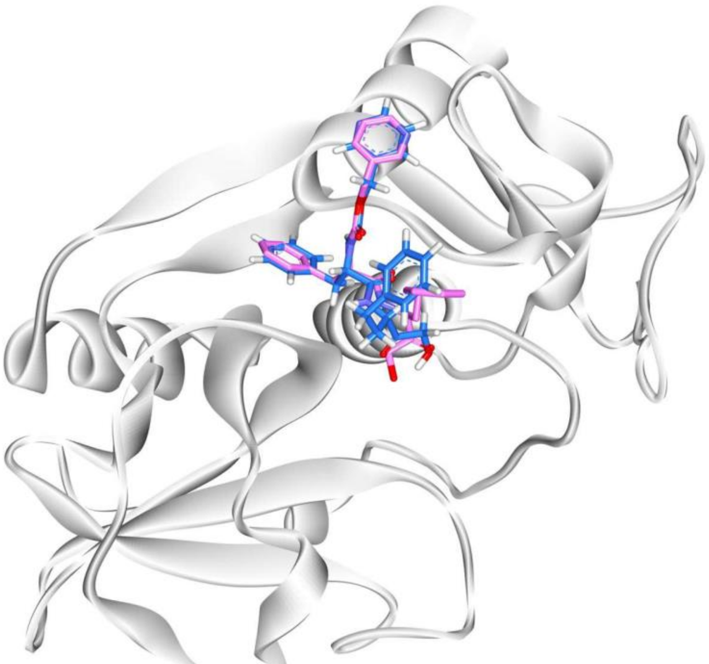

Supplementary figure 3.

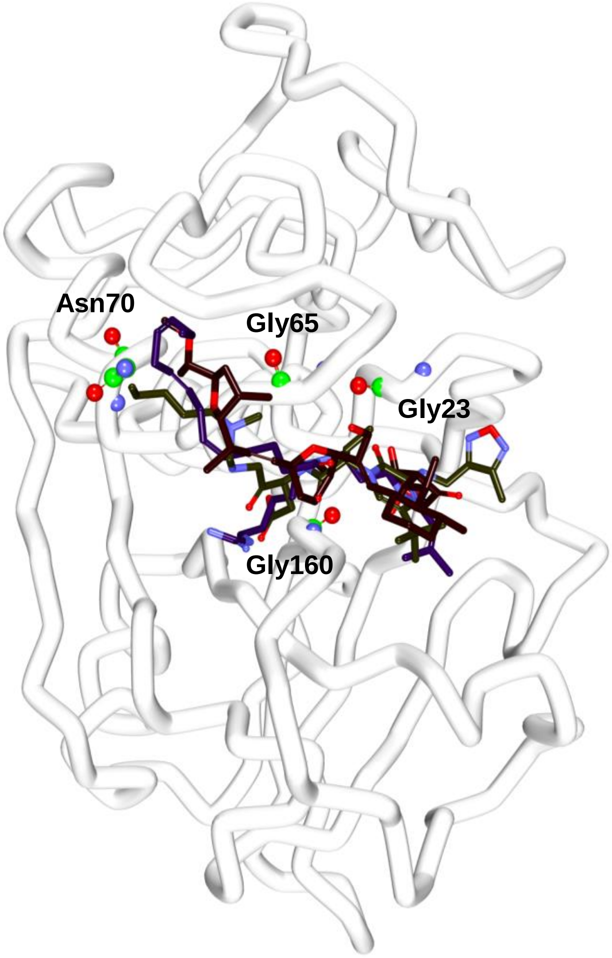

Supplementary figure 4.
